# Gallbladder Hypoplasia With Intestinal Malrotation in a Patient With Apert Syndrome: A Case Report

**DOI:** 10.1155/cris/5588836

**Published:** 2026-09-18

**Authors:** Lavanya Easwaran, Sage A. Vincent, Jose L. Diaz-Miron

**Affiliations:** ^1^ Department of Surgery, University of Colorado School of Medicine, 12631 East 17th Avenue, Aurora 80045, Colorado, USA, ucdenver.edu; ^2^ Division of Pediatric Surgery, Children’s Hospital Colorado, 13123 East 16th Avenue, Aurora 80045, Colorado, USA, childrenscolorado.org

## Abstract

Gallbladder agenesis and hypoplasia are rare diseases that are often asymptomatic but can present as biliary colic in the 5^th^ or 6^th^ decade of life. The incidence of gallbladder agenesis and hypoplasia has not been well documented in the pediatric population, and concomitant congenital anomalies can result in a complex clinical presentation. This is a case report of a 20‐year‐old male with Apert disease with a 6‐month history of chronic right upper quadrant (RUQ) pain with imaging that demonstrated both gallbladder hypoplasia and malrotation without volvulus. He underwent both a cholecystectomy and a Ladd’s procedure upon intraoperative identification of a hypoplastic gallbladder and had complete resolution of pain. The etiology of this patient’s abdominal pain cannot be conclusively identified due to the multiple procedures performed, but prior studies indicate that it may be more attributed to the gallbladder hypoplasia. This case highlights the difficulty of both gallbladder hypoplasia diagnosis and its surgical management. Gallbladder hypoplasia is exceedingly rare, and a high clinical suspicion is needed to diagnose its presence in patients with genetic predispositions for biliary disease. While there is no gold standard for its management, initial recommendations are to proceed with conservative treatment with antispasmodics before operative intervention. In the case of operative intervention, surgeons must understand the high risks of operating in these patients due to the degree of fibrosis.

## 1. Introduction

Congenital abnormalities of the gallbladder are rare and encompass a range of disorders in which the gallbladder is atretic or absent [1]. Gallbladder agenesis has a reported incidence of ~1 in 6500 [2]. It has a female gender predominance of 3:1 and is associated with other malformations, including gastrointestinal, cardiovascular, and musculoskeletal abnormalities. Similarly, gallbladder hypoplasia is another congenital biliary anomaly, which carries associations with cystic fibrosis, cholangitis, neonatal hepatitis, and biliary atresia [3]. Both gallbladder agenesis and hypoplasia are felt to be the result of abnormal in utero cystic bud development [4]. Both pathologies are typically asymptomatic and are identified incidentally on cross‐sectional imaging but can present with symptoms mimicking biliary colic [1]. Although reports of gallbladder agenesis or hypoplasia are rare in the literature, it is perhaps even more rare in the pediatric and young adult setting, as most case reports highlight patients presenting in their 5^th^ or 6^th^ decades of life [3]. Standard imaging studies can be misleading or inconclusive, making the diagnosis and management of this disease difficult [2].

This case report presents a patient with a diagnosis of Apert syndrome, a rare disease whose incidence is significantly associated with increasing paternal age and is estimated to occur in ~1 in 65,000 births with equal gender distribution [5]. Multisuture craniosynostosis, abnormal skull base development, midface hypoplasia, and symmetric syndactyly are all features of the syndrome and present with variable expressivity [6]. Patients with Apert syndrome can also present with other congenital abnormalities, including structural cardiac, gastrointestinal, and genitourinary tract anomalies [7]. Case reports and case series have also demonstrated that Apert syndrome can present with esophageal atresia, pyloric stenosis, biliary atresia, and anorectal anomalies [7–9]. Despite these varied associations, visceral dysfunction in Apert syndrome and other craniosynostosis disorders is sparsely described in current literature, requiring a high index of suspicion for diagnosis and management [10].

This case report aims to raise awareness of the clinical presentation of gallbladder agenesis or hypoplasia to guide earlier diagnosis and management.

## 2. Case Presentation

**Figure 1:**
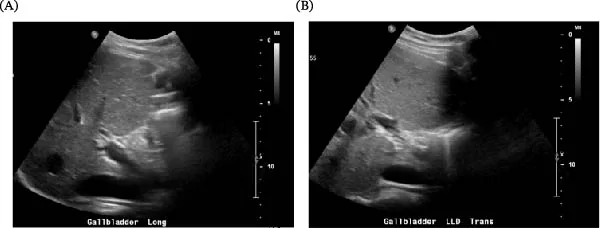
(A, B) Right upper quadrant ultrasound images with a nonvisualized gallbladder and a normal liver.

**Figure 2:**
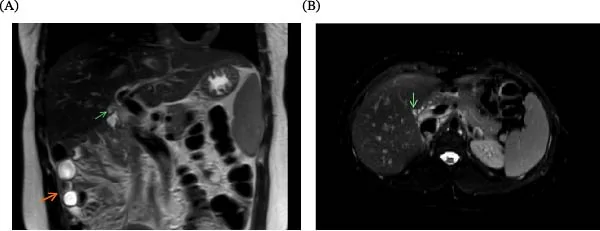
(A, B) Magnetic resonance cholangiopancreatography (MRCP) with a subcentimeter hyperintense focus in the gallbladder fossa (marked with green arrows), potentially representing a severely hypoplastic gallbladder vs. a mildly prominent cystic duct segment. (A) Malrotation seen with duodenum entirely in right upper quadrant and not crossing midline (marked with red arrow). No midgut volvulus present.

A 20‐year‐old male with history of Apert syndrome, diagnosed 10 days after birth through physical exam presentation of syndactyly, characteristic facies, and widened sagittal suture and with computed tomography (CT) of the head demonstrating bilateral coronal synostosis, initially presented to the pediatric gastroenterology (GI) clinic with complaints of 6 months of chronic right upper quadrant (RUQ) abdominal pain. He reported worsening pain postprandially with intermittent postprandial bilious emesis and chronic constipation. He had previously been managed by a primary care physician with acid blockade, which gave him inconsistent and mixed relief of symptoms. Labs demonstrated normal bilirubin levels, and a RUQ ultrasound (US) could not visualize a gallbladder (Figure 1). Magnetic resonance cholangiopancreatography (MRCP) was then performed with findings of a subcentimeter T2 hyperintense focus in the gallbladder fossa, suggesting a severely hypoplastic gallbladder vs. a prominent cystic duct segment. These findings were initially interpreted as concerning for gallbladder atresia. The MRCP also demonstrated incidental intestinal malrotation without volvulus (Figure 2). Following multidisciplinary conversations with GI and pediatric surgery teams, the patient’s chronic RUQ pain was felt to be potentially related to malrotation, and both teams agreed that further imaging with hepatobiliary iminodiacetic acid (HIDA) scan, CT, or endoscopic US was not indicated. The patient was scheduled for an exploratory laparoscopy and Ladd’s procedure.

**Figure 3:**
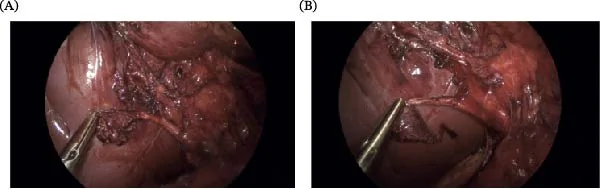
(A) Intraoperative laparoscopic images of hypoplastic gallbladder. (B) Intraoperative laparoscopic images of diminutive cystic duct.

Upon entry into the abdomen, viable intestines were present with omental adherence to the liver. This omentum was dissected off from the liver for better visualization of the porta hepatis, where a hypoplastic structure containing presumed stones was seen (Figure 3).

Dissection of the area was performed in a top‐down approach, and a small duct was noted at its base, which appeared to empty into the common bile duct. Attempts to cannulate the presumed cystic duct were unsuccessful given its small caliber; thus, intraoperative cholangiogram could not be completed. The structure was noted to have bile excretion following ductotomy, further suggesting its identity as a hypoplastic gallbladder. No other structures, such as a cystic artery, were seen entering the diminutive gallbladder. The presumed cystic duct was clipped and divided, and the gallbladder was removed without issue. A nonrotated configuration of the bowel was achieved after Ladd’s procedure, and routine appendectomy was also performed.

**Figure 4:**
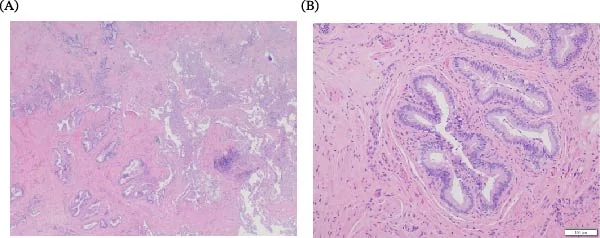
(A, B) Histological images of hypoplastic gallbladder. (A) Smooth muscle and fibroadipose tissue with a cystic region containing abundant autolyzed epithelium. (B) Numerous glands with fibrous tissue and between smooth muscle fascicles.

Final surgical pathology showed disorganized smooth muscle with biliary and focal mucinous epithelium, most suggestive of a congenital malformation in the gallbladder hypoplasia/aplasia spectrum (Figure 4). There was no evidence of inflammation or calculi in the specimen. The cystic duct margin included in the specimen had similar features with no definite dominant lumen. No vascular tissue was identified in the specimen.

The patient’s postoperative course was uncomplicated. His diet was slowly advanced, and he was able to meet all milestones for discharge on postoperative day 4. The patient was seen in the clinic 2 weeks postoperatively. He reported resolution of all abdominal pain and denied nausea or emesis. He was functioning at baseline and tolerating a regular diet with no restrictions and was advised to have additional surgical follow‐up on an as‐needed basis. On 14‐month follow‐up with the multidisciplinary team managing Apert syndrome, the patient reports doing well with no abdominal concerns.

## 3. Discussion

This case report highlights a complex differential diagnosis of chronic RUQ abdominal pain in the setting of concomitant congenital anomalies. While postprandial RUQ abdominal pain is typical of gallbladder dysfunction, imaging findings suggestive of gallbladder hypoplasia or aplasia led the medical and surgical teams to investigate alternative etiologies of the patient’s symptoms. Intestinal obstruction due to malrotation, biliary dyskinesia, and pancreatitis was among the pathologies on the differential diagnosis. Ultimately, this patient underwent an exploratory laparoscopy, Ladd’s procedure, and cholecystectomy with complete resolution of abdominal pain and intermittent bilious emesis. There remains some degree of uncertainty as to the ultimate etiology of the patient’s symptoms, given the multiplicity of surgical procedures performed.

Diagnosis of gallbladder agenesis or hypoplasia is difficult, and due to the rarity of the condition, most clinicians have a low index of suspicion for the disease, which can result in inappropriate surgical intervention. Falsely positive RUQ US studies or incorrect interpretation of nonvisualization of the gallbladder on HIDA scan could easily lead a clinician to proceed with surgical intervention, only making the diagnosis of gallbladder agenesis or hypoplasia intraoperatively [11]. Inconclusive US studies of gallbladder pathology should also alert the clinician to consider ectopic gallbladder, in which the organ can be located in areas such as the greater omentum or the retroperitoneum [3]. Surgery in this patient population does pose risks due to the anatomic variation. Hypoplastic gallbladders are frequently diminutive and fibrotic‐appearing, which also makes intraoperative identification difficult. Additionally, hypoplastic gallbladders may not present with standard cystic artery and cystic duct structures, though data on this is limited to what has been described in other case reports [12, 13]. Surgical exploration in this setting presents a high risk of damage to the liver, biliary tree, small bowel, and hepatic vessels secondary to the intensive dissection [2]. In these circumstances, it is recommended that the surgeon initially explore the abdomen to rule out an ectopic location of a normal‐sized gallbladder. Intraoperative use of indocyanine green could be helpful during surgical explorations in these settings as it has been shown to aid with visualization and identification of the biliary tree [14]. New advances in artificial intelligence (AI) could also potentially aid intraoperatively with the identification of biliary structures, though a recent systematic review of this technology acknowledges that there are currently still barriers to full implementation of AI in this manner [15].

Given the technically difficult elements of biliary dissection in this setting, it is preferred to establish a diagnosis on imaging rather than risk iatrogenic injury intraoperatively. Identification of gallbladder agenesis or hypoplasia on imaging allows clinicians to attempt conservative management first, avoiding the need for a challenging cholecystectomy. MRCP is the diagnostic method of choice due to detailed evaluation of the biliary tract, allowing for exclusion of ectopic gallbladder and accurate diagnosis of gallbladder agenesis or hypoplasia [3, 16]. Patients with gallbladder agenesis or hypoplasia can present with biliary colic symptoms, as seen in this patient. In the absence of a physical gallbladder, the pain is hypothesized to be secondary to sphincter of Oddi dysfunction, resulting in biliary dyskinesia with spasms of the sphincter [3, 17]. Most clinicians recommend treating symptomatic patients with analgesics and antispasmodics; in severe cases, some clinicians suggest sphincterotomy [3, 16, 18]. Despite these recommendations, preoperative diagnosis of gallbladder agenesis or hypoplasia remains difficult, and if exploratory laparoscopy is pursued to aid in diagnosis, current data support aborting the procedure when clear anatomy cannot be readily identified [19]. Additionally, surgeons performing exploratory laparoscopy in these cases should be prepared to discover other concomitant anomalies that may require intervention, such as concurrent intestinal malrotation, as encountered in this patient [3].

It is worth mentioning that this patient’s concurrent presentation of malrotation and gallbladder hypoplasia contributed to the diagnostic difficulty and dilemma regarding optimal treatment. While management recommendations for patients with symptomatic malrotation are well established, the treatment of asymptomatic or incidentally discovered malrotation remains controversial. In a large review of available literature in 2015, the American Pediatric Surgical Association Outcomes and Evidence‐Based Practice Committee concluded that while consideration for surgical management of asymptomatic malrotation may be considered in neonates and infants, observation is more appropriate in older patients given multiple studies demonstrating a decreased incidence of volvulus in these patients [ 20]. Another review reports a 0.6% incidence of volvulus in patients who are asymptomatic versus a rate of 15% of adhesive small bowel obstruction following surgical intervention, indicating the risks of surgery for asymptomatic patients may outweigh the benefits of volvulus prevention [21]. In the case of this patient, the constellation of symptoms, including bilious emesis, was concerning for possible intestinal obstruction, which led to the need for operative exploration.

To our knowledge, only one other similar case report has been published, highlighting a patient with both gallbladder agenesis and malrotation [11]. In this patient’s case, RUQ US reported visualization of an impacted gallbladder with sludge and small stones, as well as multiple MRCPs with conflicting reports ranging from normal to absent gallbladder. Intraoperatively, the gallbladder fossa contained only fibrous tissue, and no gallbladder was identified on pathology review of the excised tissue, consistent with agenesis. Malrotation was also confirmed intraoperatively with the duodenojejunal flexure to the right of midline; however, Ladd’s procedure was not performed in this case. The patient in this case also had resolution of all symptoms, similar to our patient represented here, indicating that the etiology of his symptoms may have been biliary colic related to gallbladder hypoplasia.

In conclusion, this case report highlights a complicated presentation of gallbladder hypoplasia with concomitant malrotation without volvulus. While the patient’s initial presentation was consistent with a biliary pathology, his ongoing bilious emesis and the demonstration of malrotation on imaging studies led to operative exploration, where a diminutive structure resembling a gallbladder was found, leading to completion of both a Ladd’s procedure and cholecystectomy. Final pathology of the diminutive structure demonstrated gallbladder hypoplasia. Although gallbladder agenesis or hypoplasia is incredibly rare, and there is no gold standard for its management, the literature suggests attempting conservative management initially and reserving operative interventions as a last resort, given the difficulty of dissection frequently encountered. In the case of rare congenital pathologies, the importance of multidisciplinary discussions cannot be overstated. Based on this, we recommend multidisciplinary collaborations and higher clinical suspicion for gallbladder agenesis or hypoplasia, especially in patients with genetic predispositions for biliary disease, such as Apert syndrome. This study is limited to a single case report, and further investigation is indicated to better understand the symptomatology and optimal management of patients with gallbladder agenesis or hypoplasia in the future.

## Funding

No funding was received for this research.

## Consent

Written informed consent was obtained from the patient for the publication of this case report and any accompanying images.

## Conflicts of Interest

Jose Diaz‐Miron is currently developing a new laparoscopic port system with MedCore technologies. The other authors declare no conflicts of interest.

## Supporting Information

Additional supporting information can be found online in the Supporting Information section.

## Supporting information


**Supporting Information** Supporting A: CARE checklist for case report.

## Data Availability

The data that support the findings of this study are available upon request from the corresponding author. The data are not publicly available due to privacy or ethical restrictions.
